# Socio economic crisis and mortality. Epidemiological testimony of the financial collapse of Argentina

**DOI:** 10.1186/1477-9560-3-22

**Published:** 2005-12-13

**Authors:** Enrique P Gurfinkel, Gerardo E Bozovich, Omar Dabbous, Branco Mautner, Frederick Anderson

**Affiliations:** 1 Cardiology and Cardiovascular Surgery Institute, Favaloro Foundation, Buenos Aires, Argentina; 2 Instituto Argentino de Diagnóstico y Tratamiento, Buenos Aires, Argentina; 3 Center for Outcomes Research, The University of Massachusetts Medical School, Worcester, MA. USA

## Abstract

**Background:**

Natural disasters, war, and terrorist attacks, have been linked to cardiac mortality. We sought to investigate whether a major financial crisis may impact on the medical management and outcomes of acute coronary syndromes.

**Methods:**

We analyzed the Argentine cohort of the international multicenter Global Registry of Acute Coronary Events (GRACE). The primary objective was to estimate if there was an association between the financial crisis period (April 1999 to December 2002) and in- hospital cardiovascular mortality, with the post-crisis period (January 2003 to September 2004) as the referent. Each period was defined according to the evolution of the Gross Domestic Product. We investigated the demographic characteristics, diagnostic and therapeutic procedures, morbidity and mortality.

**Results:**

We analyzed data from 3220 patients, 2246 (69.8%) patients in the crisis period and 974 (30.2%) in the post-crisis frame. The distribution of demographic and clinical baseline characteristics were not significantly different between both periods. During the crisis period the incidence of in-hospital myocardial infarction was higher (6.9% Vs 2.9%; p value < 0.0001), as well as congestive heart failure (16% Vs 11%; p value < 0.0001). Time to intervention with angioplasty was longer during the crisis, especially among public sites (median 190 min Vs 27 min). The incidence proportion of mortality during hospitalization was 6.2% Vs 5.1% after crisis. The crude OR for mortality was 1.2 (95% C.I. 0.87, 1.7). The odds for mortality were higher among private institutions {1.9 (95% C.I. 0.9, 3.8)} than for public centers {1.2 (95% C.I. 0.83, 1.79)}. We did not observe a significant interaction between type of hospital and crisis.

**Conclusion:**

Our findings suggest that the financial crisis may have had a negative impact on cardiovascular mortality during hospitalization, and higher incidence of medical complications.

## Background

In comparison with other Latin American nations, Argentina used to enjoy a relatively developed economy and a fair distribution of wealth until the early 1980s. During the last decade of the 20^th ^century the economy was re-engineered almost completely to create an open market economy with practically no transition. The country paid a high toll for this change, with the Gross Domestic Product experiencing a sustained decline from 1998, and unemployment rates reaching approximately 25 percent. By the end of 2001 a rapid cascade of political and economic events opened the road to deep social turmoil and economic unrest that spiraled until December 2001, when the country experienced a virtual halt of vital areas of the economy. With two more years of his period still to be completed, the president left office, and so did several others over the following weeks. Less than a month after the world learned about such unusual events, the interim president addressed the Congress to announce that the country would default from all its national and international debts. Bank deposits were seized and thousands of citizens and businesses were left bankrupt while the national currency was devaluated by almost 200% compared to the US dollar. Shortly after the crisis erupted, many found their investments and personal savings reduced by two thirds when measured in hard currency. During the year of 2002, the gross national product declined by more than 11%, and the level of unemployment blast off[1].

Several elegant and landmark epidemiological studies have established a link between trauma produced by war, terrorism, festivities, and mortality [2-5]. However, there is scarce data on the relationship between cardiac morbidity and mortality and a major economic crisis in the absence of war or natural disasters.

The Global Registry of Acute Coronary Events started in 1999 and continued throughout the crisis and the following period, thus giving us a unique opportunity to get a real time picture of the unfolding morbidity and mortality events by means of an multicenter cohort of patients whose clinical characteristics were registered with standardized methods, definitions and selection procedures. We sought to determine whether the financial crisis was associated with cardiac mortality and if medical procedures and therapies were affected by type of institution, public or private.

## Methods

Full details on the GRACE rationale and methodology have been published [6,7]. GRACE was designed to reflect an unbiased population of patients with acute coronary syndromes, irrespective of geographic region. Currently, 104 hospitals located in 14 countries (Argentina, Australia, Austria, Belgium, Brazil, Canada, France, Germany, Italy, New Zealand, Poland, Spain, United Kingdom, and the United States) are participating in this observational study. A broad range of hospitals was chosen based on the availability of differing facilities for care, including presence of on-site cardiac catheterization, number of acute care beds, and type of practice setting, such as teaching/non-teaching, and tertiary versus community hospital. This was done to establish a representative rather than a select sample of patients in the community at large. A standardized data collection form was used to collect information on demographics, symptoms, medical history, clinical, electrocardiographic and laboratory data, and in-hospital treatment and outcomes. The data forms were forwarded to the core laboratory (Premier Research, Philadelphia, PA) where, following a review of case records for completeness and face validity, the data were entered by scanning the forms directly into the database. Once entered, the data were sent to the international coordinating center for GRACE (Center for Outcomes Research, University of Massachusetts Medical School, Worcester, MA, U.S.A.) for analysis.

### Study population

For the purposes of this analysis we restricted our study sample to patients enrolled in centers in Argentina between April 1999 and September of 2004, including individuals who had an admission diagnosis of acute coronary syndrome (ST segment elevation and non-ST segment elevation myocardial infarction or unstable angina). The seven sites participating in Argentina, were analyzed all together, and also stratified according to their particular profile: Private hospitals (for profit centers, appropriate 24 hours a day interventional facilities, teaching and non-teaching centers), and public hospitals (free of charge services, no interventional facilities available 24 h a day). The period of time examined was divided into the crisis period, which was delimited from April 1999 to December 2002, and the post crisis period, which encompassed the time from January 2003 to September 2004. To define each period we used indicators published by the Census Bureau. We considered the beginning of the negative slope of the gross domestic product curve as the start of the crisis period, which lasted until the domestic product experienced a sustained increase over a full trimester [1].

### Clinical endpoints

The primary endpoint of the study was in-hospital all-cause mortality. The secondary endpoint was non-fatal-myocardial infarction defined by the presence of at least one positive increment of cardiac biochemical marker of necrosis (in case of those in whom myocardial infarction was the index diagnosis) plus chest pain prolonged more than 10 minutes, or new ST-segment deviation seen after the index or qualifying electrocardiogram.

### Statistical analysis

Summary statistics are presented as frequencies and percentages. Comparisons between groups were made using two-tailed Wilcoxon rank-sum test for continuous variables and the chi-square or Fisher's exact test for categorical variables. Odds ratios and accompanying 95% confidence intervals were computed to evaluate the effects of the crisis on hospital mortality and morbidity. The standard error for the calculation of the 95% confidence intervals for the odds ratios was calculated by means of the Wald formula, and the errors were handled independently for the different time points. Similar analyses were conducted for public and private hospitals separately. All tests were double sided and considered statistically significant at p-value < 0.5. Statistical analyses were conducted with the SAS V. 9.1 software (SAS Institute, Cary, NC).

## Results

### Study patients and baseline characteristics

Of the 44,991 acute coronary syndrome patients admitted in the global registry, 3220 patients were enrolled in Argentina. The number of patients younger than 65 years old was 1527 (47%), representing the proportion of the population normally expected to be economically active. The remainder 1693 (53%) were older than 65. A final diagnosis of ST-segment-myocardial infarction was made in 1179, and 2041 qualified as unstable angina / non-ST-segment elevation myocardial infarction. Female gender represented 30% (n = 1012) of patients. Baseline characteristics comparing patients during and after crisis did not differ significantly (Table 1). The proportion of patients with ST segment deviation on the admission ECG was identical, but the proportion of patients with positive cardiac markers during hospitalization was higher during crisis (54%, n = 1212 v. 48%, n = 472).

**Table 1:** Baseline characteristics comparing patients during and after crisis.

	Crisis median (P 25, P 75)	After Crisis median (P 25, P 75)	p value
Male gender (%)	1542 (69.6)	666 (68.5)	0.5480
Age (years)	66.1 (56, 75)	65 (56, 74)	0.4742
Weight (kg)	77 (69, 87)	79 (68, 88)	0.8390
Height (cm)	168 (161, 174)	170 (162, 174)	0.1501
Heart rate (b/min)	75 (64, 86)	74 (63, 88)	0.7656
SBP	140 (120, 160)	135 (120, 160)	0.3342
DBP	80 (70, 90)	80 (70, 90)	0.2316
Creatinine (mg/dl)	1.1 (0.9, 1.3)	1.1 (0.9, 1.3)	0.7528
Cholesterol (mg/dl)	200 (170, 230)	200 (167, 231)	0.9997

Values are expressed in median, 25^th^, and 75^th ^percentiles. The data did not differ significantly from both periods observed. SBP: Systolic blood pressure. DBP: Diastolic blood pressure.

**Figure 1:**
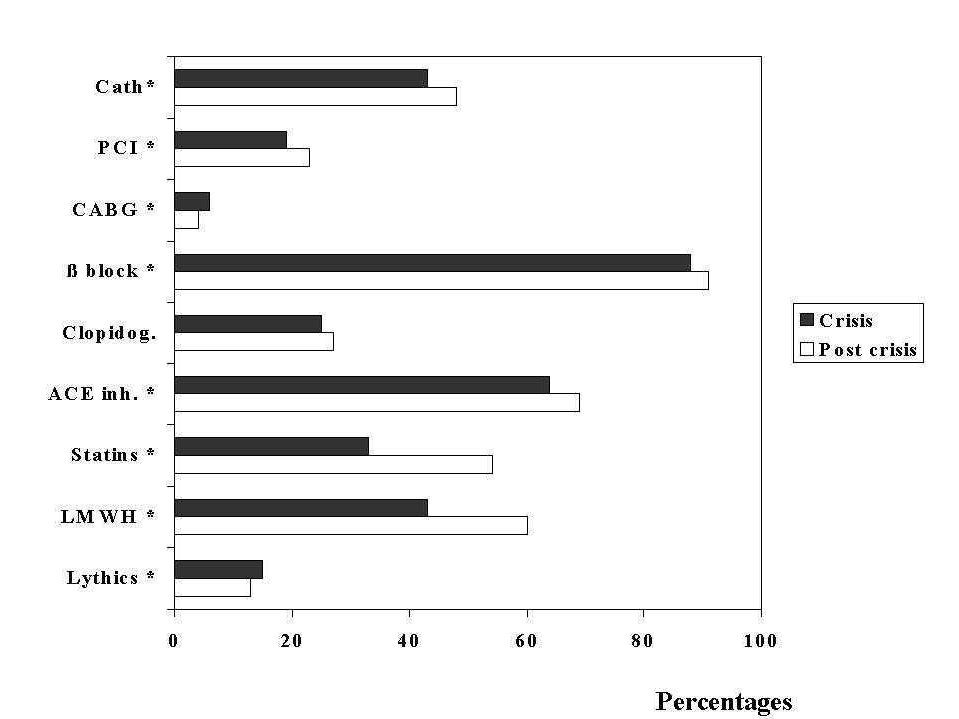
Cath: Catheterization during hospital stay. PCI: Percutaneous coronary interventions. CABG: Coronary artery by pass grafting. β block: Use of beta blockers. Clopidog. : Use of clopidogrel. ACE inh. : Use of angiotensin converting enzime inhibitors. LMWH: Low molecular weight heparin. The overall pattern shows a lower utilization of every intervention and key evidence based medicine during the crisis, which may suggest a poorer quality of care. Asterisks indicate a p value of less than 0.05.

**Figure 2:**
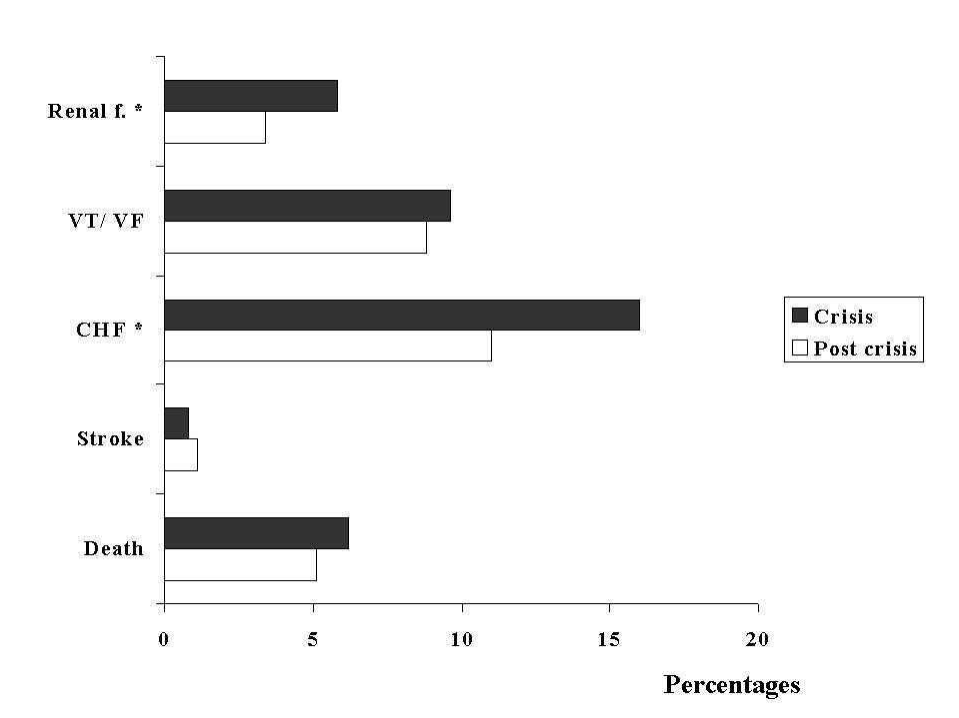
CHF: Congestive heart failure. VT/VF: Ventricular tachycardia/ventricular fibrillation. Renal f.: Acute renal failure. The incidence of medical complications was higher during the crisis, in the absence of a higher risk profile at baseline, suggesting an influence by poorer quality of care alone or combined with other factors such as impaired social support or depression. Asterisks denote a p value of less than 0.05.

During the crisis, fewer patients underwent diagnostic angiography (23%, n = 500 Vs 26%, n = 249), and related to this a lower proportion was referred to angioplasty (19%, n = 423 Vs 23%, n = 222), with a larger fraction undergoing coronary bypass surgery (5.9%, n = 131 Vs 4.1%, n = 40). We also observed some evidence of lower adherence to interventions of proven efficacy during the crisis, as shown by a lower proportion of patients receiving aspirin (96%, n = 2164 v.98%, n = 950), angiotensin converting enzyme inhibitors (64% n = 1438 Vs 69%, n = 672), and low molecular weight heparin 43%, n = 950 Vs.60%, n = 585) (Figure 1). Despite the fact that serum creatinine levels and clinical profile were similar for each period, we observed a higher incidence of renal failure during the crisis (5.8%, n = 139 Vs 3.4 %, n = 33), which may indicate a less efficient medical management during hospitalization. (Figure 2)

**Figure 3:**
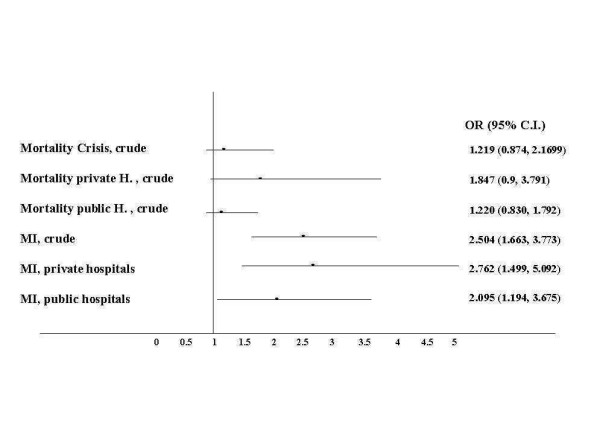
Forest plot of the odds ratios form mortality and the related 95% confidence intervals. On visual inspection there is an apparent clear trend for an association between the crisis period and in-hospital cardiac mortality (primary endpoint) and myocardial infarction (secondary endpoint). The association is borderline with statistical significance, with relatively narrow confidence intervals suggesting precise estimates, especially for the overall population and public hospitals (H). The trend seems to be more significant for patients admitted to private hospitals.

The incidence of death was higher in the crisis period. (6.2%, n = 139 Vs 5.1%, n = 50), with a crude OR of 1.2 (95% C.I. 0.87, 1.70) (Figure 3). We also observed a consistent trend for a higher incidence of in hospital complications during the crisis, including myocardial infarction {OR 2.504 (95% C.I. 1.663, 3.773)}, congestive heart failure (16% Vs. 11%, p value < 0.01), and sustained ventricular tachycardia (3.4%, n = 75 Vs 2.9%, n = 28). (Figure 2).

**Figure 4:**
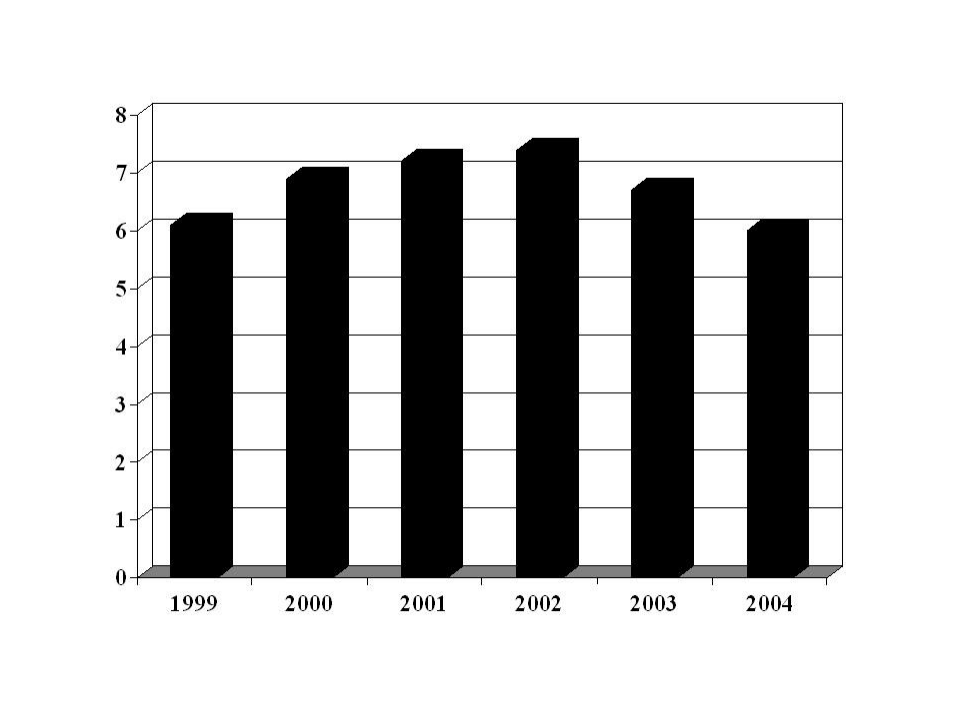
Cumulative incidence of death for individual calendar years 1999–2004.

In order to explore a potential effect of time dependency over outcomes, we calculated separately the cumulative incidence of mortality for every calendar year. (Figure 4) The highest mortality incidence was 6.1% in 1999, and peaked at 7.4% in 2002, to finally decrease to 6% in 2004. (Figure 4)

### Interventions and outcomes by type of hospital

We observed some indirect data suggestive of a major shift in accessibility to medical care as indicated by the proportion of patients admitted to public hospitals, which in the Argentine GRACE cohort increased from 47% to 60%.

The types and frequency of interventions shared some aspects in common between public and private sites, and was different in several others. During the post crisis period we observed an increase in the proportion of angiography and percutaneous coronary interventions, and also in the proportions treated with aspirin, statins, and low molecular weight heparin (Table 2). Of note, following the crisis the median time delay to percutaneous intervention decreased noticeably in private centers from 50 h (interquartile range 126.5) to 25 h (interquartile range 60.4), and in public sites from 190 h (interquartile range 392) to 27 h (interquartile range 172.1), both p values < 0.01. We did not observe a statistically significant difference in median time to thrombolysis neither for private sites {30 h v. 33 h (interquartile ranges 35 and 80)}, nor for public ones {59 h v. 40 h (interquartile ranges 85 and 65 respectively)}. On the other hand, during the post crisis time we observed a decline in the proportion of patients undergoing coronary by pass surgery, particularly in private sites (Table 2).

**Table 2:** Hospital Profiles, Diagnosis, procedures and Medications

	Private Hospitals			Public Hospitals		
	Crisis	After crisis		Crisis	After crisis	
	Frequency (%)	Frequency (%)	p value	Frequency (%)	Frequency (%)	p value

STEMI	411 (35)	150 (39)		443 (42)	175 (30)	
NonSTEMI / UA	771 (65)	237 (61)		621 (58)	412 (70)	
Cardiac Catheterization	616 (52)	241 (62)	0.0006	343 (33)	219 (38)	0.0382
PCI	356 (30)	168 (43)	<0.0001	67 (6.4)	54 (9.2)	0.0373
CABG	115 (9.8)	28 (7.3)	0.1355	16 (1.5)	12 (2.1)	0.4412
Echocardiography	774 (66)	217 (57)	0.0006	365 (35)	219 (38)	0.2018
PA catheter	104 (8.8)	17 (4.4)	0.0050	29 (2.7)	17 (2.9)	0.8377
Invasive Respiratory Assistance	139 (12)	30 (7.8)	0.0263	74 (7.0)	30 (5.2)	0.1433
*In hospital*	*medication*					
Aspirin	1137 (96)	379 (98)	0.1001	1027 (97)	571 (97)	0.4044
Beta blockers	1049 (89)	358 (93)	0.0120	928 (87)	524 (89)	0.2213
ACE inhibitors	740 (63)	265 (69)	0.0502	698 (66)	407 (69)	0.1566
Clopidogrel	429 (37)	205 (53)	<0.0001	114 (11)	58 (10)	0.5095
Statins	462 (39)	216 (56)	<0.0001	269 (26)	309 (53)	<0.0001
Low molecular weight heparins	626 (54)	291 (76)	<0.0001	324 (31)	294 (50)	<0.0001
Thrombolytics	142 (12)	33 (8.6)	0.0586	203 (19)	88 (15)	0.0373

Values are expressed as numbers and proportions.

STEMI: ST-segment myocardial infarction, non-STEMI / UA: non-ST-segment myocardial infarction and Unstable Angina, PCI: percuntaneous coronary intervention; CABG: Coronary artery by-pass grafting; PA: Pulmonary artery catheter

In a similar trend, we found that the vast majority of adverse events detected during the crisis period, such as myocardial infarction, congestive heart failure, and death, begun to decrease from January 2003 to September 2004, the period of time when the National Census Bureau detected a recovery of the Gross Domestic Product.

The odds for myocardial infarction were higher during the crisis in private hospitals {OR 2.76 (95% C.I. 1.5, 5.1)} (Figure 3). In a stratified analysis, the OR for mortality during the crisis appeared to be higher for patients admitted to private centers than to public ones {1.85 (95% C.I. 0.9, 3.79) Vs 1.22 (95% C.I. 0.83, 1.79)}(Figure 3).

## Discussion

### Statement of principal findings

Our study provides some evidence that there may be an association between the financial and institutional collapse of Argentina and increased in-hospital cardiovascular morbidity and mortality. The link between extraordinary circumstances and increased cardiac mortality has been previously reported. A significant increase in the number of cardiac deaths was observed on the same day of major earthquakes that affected Los Angeles and Athens [8,9]. Also, the socio-economic difficulties experienced by Russia following the collapse of the Soviet Union have been associated with a marked decrease in the crude life expectancy both for males and females [10]. Albeit, no specific information on cardiac mortality rates due to coronary artery disease in Russia is available for that specific period.

### Strengths of the study

The Argentine case is unique in that a major socio-economic collapse occurred in the absence of any natural disaster or war. GRACE provides a useful tool to assess in a standardized, structured manner, the diagnostic and therapeutic approaches performed in a representative cluster of hospitals throughout the crisis and following it. Our observations are intriguing, and pose questions on the mechanisms underlying the increased odds of mortality during the crisis compared to the post crisis period. We analyzed several mechanisms that may be responsible for the worsened outcomes during the crisis period: Differences in baseline clinical risk, in medical interventions, type of hospital, social and psychological factors, bias and chance.

### Baseline clinical risk

We did not observe any significant differences between the crisis and post-crisis period regarding the main demographic characteristics such as age, gender, prior coronary artery disease, co morbidities and Killip class on admission. The overall clinical profile is similar to other cohorts elsewhere for the same period [6].

### Differences in medical interventions

We anticipated an association between the crisis and access to medical care because of a direct effect on access to technology and imported medical supplies. Our observations provide some evidence to support the presumption that the crisis may have affected the quality of care. On one hand, patients enrolled in the registry were consistently treated according with the guidelines and in a similar manner compared to other regions [11]. The proportion of patients receiving aspirin, beta-blockers, ACE inhibitors and statins increased progressively over the years and no relevant alterations were observed in the crisis period. Further, the proportion of patients undergoing percutaneous coronary interventions or by pass surgery followed the international trends and guidelines which appeared between periods [12,13]. On the other hand, time to invasive interventions was several fold longer during the crisis both for public and private hospitals. This may reflect a limited supply of tools that were by most produced outside the country and priced in hard currency. Also, as shown in figure 1, the proportion of patients treated with relatively expensive medications such as low molecular weight heparin and statins was significantly lower during the crisis. We also found a higher proportion of congestive heart failure during the crisis period. It could be speculated that this was related to a lower quality of care as reflected by time delay to invasive procedures as stated before, but it could also be the consequence of other factors not measured by our study. Such factors may to some extent be responsible for the inter-regional variations in outcomes of populations that appear to be otherwise similar [14-16].

### Type of institution

Another factor that could have influenced the outcomes is an increased burden of medical care on the public system. The Argentine economic phenomenon has been called a "middle class crisis", namely of those who would normally gain access to health insurance through employment or, for small business owners and entrepreneurs, as an out of pocket expenditure. Approximately 20 million people out of a total country population of 37 million are no longer covered by neither the private sector nor a union-run mandatory health insurance, which represents a huge overload for the network of public hospitals [17]. Public hospitals in Argentina suffer form chronic shortage of funding, inadequate distribution of staffing, and have limited capabilities to provide high tech, round the clock care, as reflected by the relatively low proportion of patients undergoing invasive procedures and revascularization. The sudden increase in the demand of medical services posed by the abrupt transfer of thousands of patients from the private system was not accompanied a proportional increase in budget or staff, thus making it likely that the services provided were insufficient [16].

### Social support and psychological factors

The association between the crisis period and increased in-hospital cardiac mortality could be explained by alterations in socio-economic factors or social support, both variables that were not directly measured by the registry. Socio-economic status has been used as a surrogate marker of a much complex matrix called social support. Several studies have suggested that a meaningful impairment in the quality and width of social support can be associated with higher mortality rates, both from cardiac and non cardiac causes [18,19]. It is possible that the enormous stress produced by the loss of savings, investments, and jobs yielded a proportional increase in psychological stress and sense of lack of social support, with dire consequences for the outcome of acute coronary events. It is also likely that several social covariables interacted at the same time to yield an effect on outcomes.

### Time dependency

It may be argued that our observations may be due solely to the availability of better treatment modalities over time. As shown in figure 3, this appears not to be the case. The crude cumulative incidence of death was 6.1% in 1999, then increased to 7.2% in 2001; 7.4% in 2004 and then decreased to 6% in 2004. Although we can't rule out completely the influence of new guidelines and better therapeutic options over time, the breakout analysis of annual mortality seems to support our main findings.

### Weaknesses

Our analysis is exposed to a potential source of selection bias by the definition of each time period. In the absence of a major natural disaster, disease outbreak or war, it can be argued on the accuracy on our definitions on when the crisis started and when it ended. For that matter, we considered data published by the Census Bureau regarding the National Gross Domestic Product and industrial indicators and unemployment rates, and selected the nadir of the adjusted Gross Domestic Product curve as the onset of the crisis, and the first trimester that showed a sustained increase in the Gross Domestic Product as the arbitrary end of the financial crisis. This is subject to bias in itself and alternative definitions may have yielded different results. Nevertheless, we feel confident in that our definitions are solid and based on hard economic indicators instead of political signs or personal interpretations that are vulnerable to subjective perceptions. There is a consistent match between the evolution of the gross domestic product and other indicators such as the investment indexes, public works and private investments in real estate and construction [1].

Another limitation to our conclusions is that GRACE was not specifically designed to provide information on socio economic status or social support, which would be alternative exposures of interest in the scenario of a deep financial crisis. We considered the broad term "crisis" as the exposure of interest, so we must acknowledge that the mechanisms responsible for our observations are to some extent speculative. Also, the study was not powered to detect strength of association between exposures and mortality for a specific region or country.

Even in the absence of a formal level of statistical significance, the odds ratios appear to consistently point in thee direction of worse outcomes during the crisis. (Figure 3)

## Conclusion

This study provides evidence suggestive of an association between a dramatic socio-economic event and increased cardiac mortality. The spike in mortality rates that we observed was striking and above the expected death rates according to prior projections from the Ministry of Health [20,21]. We observed a consistent trend to worse outcomes during hospitalization, thus indicating an association between the financial crisis and cardiac morbidity and mortality.

## Competing interests

The GRACE study is supported by an unrestricted grant from Sanofi-Aventis to the Center for Outcomes Research, University of Massachusetts Medical School.

Sanofi-Aventis had no involvement in the collection, analysis, and interpretation of data; in the writing of the manuscript; or in the decision to submit the paper for publication. The design, conduct, and interpretation of GRACE are undertaken by an independent steering committee.

The authors have no conflicts to declare according to the Thrombosis Journal (TJ) Declaration of Competing Interest form. The Corresponding Author has the right to grant on behalf of all authors and does grant on behalf of all authors, an exclusive license on a worldwide basis to the TJ Publishing Group Ltd and its Licensees to permit this article (if accepted) to be published in TJ editions and any other TJ products to exploit all subsidiary rights, as set out in the TJ license conditions. All authors have read and approved this manuscript.

## Authors' contributions

We thank the physicians and nurses participating in GRACE. The complete list of GRACE Investigators can be found at . EPG and GEB conceived and designed the study and wrote the manuscript. OD and FA contributed to study design and performed statistical analysis and reviewed the manuscript and BM supervised the study.

### GRACE Scientific Advisory Committee

Keith A.A. Fox, Joel M. Gore (GRACE Co-Chairs); Kim A. Eagle, Philippe Gabriel Steg, (GRACE Publication Committee Co-Chairs); Giancarlo Agnelli, Frederick A. Anderson, Jr, Álvaro Avezum, David Brieger, Andrzej Budaj, Marcus D. Flather, Robert J. Goldberg, Shaun G. Goodman, Christopher B. Granger, Dietrich C. Gulba, Enrique P. Gurfinkel, Brian M. Kennelly, Werner Klein, José López-Sendón, Gilles Montalescot, Frans Van de Werf.

**Table 3:** Major adverse events in public and private hospitals.

	Private	Hospitals		Public	Hospitals	
	Crisis	After Crisis		Crisis	After Crisis	
	Frequency (%)	Frequency (%)	p value	Frequency (%)	Frequency (%)	p value

Recurrent Ischemia	257 (22)	60 (16)	0.0063	304 (29)	169 (29)	0.9619
Myocardial infarction	96 (8.2)	12 (3.1)	0.0007	59 (5.6)	16 (2.7)	0.0090
CHF	143 (12)	29 (7.5)	0.0111	215 (20)	74 (13)	<0.0001
Cardiogenic Shock	38 (3.2)	12 (3.1)	0.9014	42 (4.0)	26 (4.4)	0.6427
Ventricular Fibrillation	56 (4.8)	12 (3.1)	0.1699	82 (7.7)	45 (7.7)	0.9824
Ventricular Tachycardia	39 (3.3)	6 (1.6)	0.0701	36 (3.4)	22 (3.8)	0.6726
Renal Failure	75 (6.4)	15 (3.9)	0.0688	54 (5.1)	18 (3.1)	0.0654
AV block	35 (3.0)	7 (1.8)	0.2246	41 (3.9)	12 (2.1)	0.0542
Stroke	4 (0.3)	2 (0.5)	0.6408	13 (1.2)	9 (1.5)	0.6553
In-Hospital Death	50 (4.2)	9 (2.3)	0.0897	89 (8.4)	41 (7.0)	0.3109

CHF: Congestive heart failure, AV Block: Atrial Ventricular Block. Myocardial infarction rates significantly decreased over time, as life threatening arrhythmias such as ventricular tachycardia, atrio/ventricular blockade, renal failure, and in-hospital death.
